# Evaluation of the Food and Drugs Authority, Ghana Regulatory Review Process: Challenges and Opportunities

**DOI:** 10.1007/s43441-022-00478-x

**Published:** 2022-11-09

**Authors:** Mercy Owusu-Asante, Delese Mimi Darko, Kwame Dei Asamoah-Okyere, Samuel Asante-Boateng, Adem Kermad, Stuart Walker, Sam Salek

**Affiliations:** 1 School of Life and Medical Sciences, University of Hertfordshire, Hatfield, UK; 2 Food and Drugs Authority, Accra, Ghana; 3 Centre for Innovation in Regulatory Science, 70, St Mary Axe, London, EC3A 8BE UK; 4 Institute of Medicines Development, Cardiff, UK

**Keywords:** FDA Ghana, Regulatory review models, Good review practices, Regulatory timelines, Quality decision-making practices, WHO GBT

## Abstract

**Purpose:**

This study aimed to assess the current regulatory review process of the food and drugs authority (FDA) Ghana by identifying key milestones, target timelines, good review practices and quality decision-making practices and evaluating the overall regulatory performance from 2019 to 2021, as well as the challenges and opportunities for improvement.

**Methods:**

The FDA Ghana representatives completed the optimising efficiencies in regulatory agencies (OpERA) questionnaire, including data identifying the milestones and overall approval times for all products registered by the FDA Ghana from 2019 to 2021.

**Results:**

Of the new active substances approved from 2019 to 2021, 91% were biologicals processed by full or abridged reviews pathways. Timelines for these reviews were within authority targets but were longer compared with generics. Of generics approved from 2019 to 2021, 97% were pharmaceuticals processed by the full review pathway, with timelines within authority targets and shorter compared with new active substances. Regardless of the review model used, approval times for new active substances increased from 84 to 355 calendar days 2019–2021 due to the impact of the pandemic. Guidelines, standard operating procedures and review templates were in place and the majority of indicators for good review practices were implemented. Several quality decision-making practices were implemented, although currently there is not a systematic structured approach.

**Conclusion:**

The FDA Ghana monitors regulatory performance and currently meets its target timelines. To achieve World Health Organization Maturity Level 4 status, an electronic tracking system, benefit-risk assessment framework and template and the publication of assessment reports are recommended.

## Introduction

### Ghana National Medicines Regulatory Authority

Medical products, which include medicines, vaccines and medical devices, form a core component of a national healthcare system. Ensuring the availability of high-quality, safe and effective medical products through the establishment of effective and efficient national medical regulatory authorities (NMRAs) is a national responsibility to protect public health and safety [1]. The food and drugs authority (FDA) Ghana is the National Medicines Regulatory Authority legally mandated by Parts 6, 7 and 8 of the Public Health Act 2012 (Act 851) to safeguard the safety, quality and efficacy of medical products in Ghana. The FDA Ghana vision is to “protect the health and safety of people in Ghana and to be a global centre of excellence for food and medical product regulation” [2, 3].

In West Africa, the FDA Ghana is respected by other NMRAs, as a result of its regulatory standing in the region. Currently in Africa, the NMRAs in Ghana, South Africa, Tanzania, Nigeria and Egypt are the only agencies to have achieved the World Health Organization Global Benchmarking Tool (WHO GBT) maturity level 3. On a scale of 1 to 4, the WHO GBT maturity level measures how stable, well-functioning and integrated a country’s regulatory systems performs. The common regulatory functions of an NMRA are registration and marketing authorisation, regulatory inspection, licensing of manufacturing and storage facilities, post-market surveillance, vigilance, quality control and clinical trials oversight. It is the case in most countries that medical products are first registered before they can be made available to patients [4, 5].

Ghana, one of 15 countries in West Africa, has a population of about 31 million, with a median age of 21.5 years and a life expectancy at birth of 64.1 years [6–8]. The Food and Drugs Authority was established as the food and drugs board (FDB) in 1997, following the enactment of the food and drug law (PNDCL 305B) in 1992. The FDB operated as an authority of the Ministry of Health in Ghana to regulate medicinal products for human and veterinary use, medical devices and diagnostics as well as food. Following the establishment of the FDB, the authority was transformed into the food and drugs authority (FDA) upon the enactment of the Public Health Act 2012 (Act 851) [3].

A robust NMRA supports the national healthcare system by providing safe, high-quality and effective medicines to patients, and thus it is imperative that the FDA Ghana undergoes routine performance evaluation to ascertain its effectiveness and efficiency in discharging its mandates [9, 10]. The WHO GBT has been used to assess NMRAs for regulatory system strengthening, and it is expected that when all the benchmarks are achieved and maintained, the regulatory capacity of an NMRA will be enhanced to deal with health emergencies, including pandemics [11]. The GBT evaluates the overarching national regulatory systems, which include registration and marketing authorisation, market surveillance and control, regulatory inspection, vigilance, licensing establishments, clinical trial oversight, laboratory testing, and National Regulatory Authority lot release [12].

The aim of this study was to evaluate the current regulatory review process of the FDA Ghana with the view to identifying the challenges and opportunities for improvement.

### Objectives

The objectives of this study were to

1. Assess the current regulatory review process of the FDA Ghana
2. Identify the key milestones and target timelines achieved in the review process
3. Evaluate the overall performance for the review models and different product types approved in Ghana during the period 2019–2021
4. Assess authority compliance with good review practices and quality decision-making practices employed in the review process
5. Identify the challenges and opportunities for an enhanced regulatory review process in Ghana, with a view to expediting patients’ access to life-saving medicines

## Methods

### Ethical Approval

The study was approved by the Health, Science, Engineering and Technology ECDA, University of Hertfordshire, United Kingdom.

The type of study conducted and presented in this report did not require ethical approval from the FDA Ghana. Permission was granted by the FDA Ghana for data collection and their subsequent publication.

### Study Rationale

Since the regulatory review process and performance of Ghana FDA had not been evaluated to date, this study would form a baseline for the authority moving forward.

### Data Collection Process

The review processes and practices within the FDA Ghana were assessed using the optimising efficiencies in regulatory agencies (OpERA) questionnaire developed by the centre for innovation in regulatory science (CIRS) for the assessment of regulatory review processes in the emerging economies [21]. This questionnaire is a unique regulatory-strengthening tool that enables all critical information necessary to assess a regulatory authority’s performance to be documented systematically. [13]. It can be utilised to monitor regulatory performance, enable comparisons with other regulatory authorities in order to evaluate good regulatory practices as well as to encourage the systematic monitoring of regulatory processes. The questionnaire was completed by senior assessors of the FDA Ghana and verified by the responsible Directors and agreed by the Chief Executive Officer.

The questionnaire consists of five parts:

- *Part 1: Organisation of the agency* This documents information on the structure, organisation and resources of the authority.
- *Part 2: Types of review models* This identifies the different types of review models (verification, abridged, full) used to assess applications for marketing authorisation, including the extent to which applications are evaluated with regard to how an authority might rely on the results of assessments and reviews carried out by a reference authority.
- *Part 3: Key milestones in the review process* This captures information on the key milestones in the review process as well as providing a validated process map, which includes receipt of the dossier, validation and screening, questions to the sponsor and the final decision on approval or refusal of a product for registration. Data were collected for new active substances (NASs) and generics during the period 2019–2021.
- *Part 4: Good review practices (GRevP)* This enables the evaluation of how quality is built into the regulatory review process by examining activities that have been adopted to improve the consistency, transparency, timeliness and competency of the review process.
- *Part 5: Quality decision-making practices* This explores information on the quality of the decision-making practices and whether the authority has measures in place to ensure that quality decisions are made about the data obtained during the registration process.

## Results

The results are presented in six parts: Part 1—Organisation of the authority; Part 2—Types of review models; Part 3—Key milestones in the review process; Part 4—GrevP: building quality into the regulatory process; Part 5—Quality Decision-Making Practices; and Part 6—Concluding observations: a summary of the FDA Ghana challenges and opportunities in regulatory review.

### Part 1—Organisation of the FDA Ghana

The FDA Ghana is an authority of the Ministry of Health. It has a staff capacity of 683 across all the 16 regions of the country. The authority has 26 reviewers comprising 25 pharmacists and one scientist, who holds a PhD in Pharmaceutical and Biological Chemistry. These reviewers are responsible for the scientific assessment of marketing authorisation applications.

The FDA regulates medicinal products for human and veterinary use as well as medical devices and in vitro diagnostics. The authority’s scope of activities includes registration and marketing authorisation, market surveillance and control, regulatory good manufacturing practice (GMP) inspection, vigilance, licensing establishments, clinical trial oversight and laboratory testing.

The authority sets target timelines for the scientific assessment of applications as well as for the overall timeline for the review and decision of such applications. A Certificate of a pharmaceutical product (CPP) is a requirement only for products manufactured outside the country and must be provided before authorisation is issued. Questions to sponsors are batched at fixed points in the review procedure.

In addition, the authority recognises medical urgency as a criterion for accelerating the review process for qualifying products. Quality, safety, and efficacy are reviewed sequentially for generics since each assessor has been equipped with the technical expertise to conduct full assessment for each generic application. In the case of NASs, quality, safety and efficacy are reviewed in parallel, since assessors have some limitations with regard to the specialised expertise required to conduct full assessment; the different modules of the dossier for NASs are therefore reviewed in parallel by different assessors who have the different requisite expertise. Price negotiation is not considered as part of the review and authorisation process. For sample testing, the focus is on checking quality in the marketplace; therefore, it does not delay decisions on marketing authorisation applications. The authority recognises the value of continuous quality improvements in increasing transparency, improving the overall consistency and predictability of the regulatory process. As part of its quality management system, the authority has adopted several quality improvements tools to monitor and improve the quality of its review process.

Standard operating procedures have been implemented as part of measures to enhance the quality of the process, whilst assessment templates are used to standardise the format and content of written reports. Transparency with stakeholders is central to the overall regulatory process at the FDA Ghana. Application fees are charged based on the type of marketing authorisation application (NASs and generic medicines). Applicants are encouraged to contact the Authority (via telephone or email) during product development, pre-submission and assessment with the possibility of meetings where necessary. The authority does not, however, charge a fee to provide scientific advice.

### Part 2—Types of Review Models

The FDA Ghana carries out three types of established regulatory reviews, namely verification, abridged and full. Within each review category, there is a consideration for an additional priority/fast track review application when the need for rapid assessment is required for patients’ access to medicines.

#### A Verification

Review is applied based on the recognition of an authorisation by a reference or benchmark authority such as the WHO. The verification process is used to validate the status of the product and ensure that the product for local marketing conforms to the authorised product. The letter of authorisation from the WHO prequalification programme is accepted by the FDA Ghana as evidence of a positive WHO prequalification. The dosage form, strength, ingredients, indications, dosage, warnings, and precautions must be identical to the authorised product. A completed dossier in the common technical document (CTD) format, including data for all modules must be submitted.

#### An Abridged

Review is applied on the pre-requisite that the product has been previously approved by a stringent regulatory authority such as the United States Food and Drug Administration (US FDA), United Kingdom Medicines and Healthcare products Regulatory Agency (UK MHRA), Health Canada or those reviewed by the European Medicines Agency (EMA) centralised registration procedures. An abridged assessment is carried out in relation to the benefit-risk assessment of the product under local conditions. In these reviews, the dosage form, strength, ingredients, indications, dosage, warnings and precautions must be identical to the authorised product, including the manufacturing site/lines and a complete dossier in the CTD format, including identical data for all modules must be submitted.

#### A Full

Review is carried out by the authority in all other situations since it is capable of carrying out a full assessment of quality, pre-clinical (safety), and clinical (efficacy) data. Information on prior registration elsewhere may be a pre-requisite to final authorisation and the dosage form, strength, ingredients, indications, dosage, warnings and precautions must be identical to the authorised product. A completed dossier in the CTD format including data for all modules must be submitted.

#### Priority/Fast Track

Review applications, where there is a need, are considered within the same category of applications. A rapid assessment is carried out to obtain pharmacological, marketing/commercialisation, pharmacovigilance and clinical trials information. A completed dossier in the CTD format, including full data for all modules must be submitted.

### Part 3—Key Milestones in the Review Process

**Figure 1:**
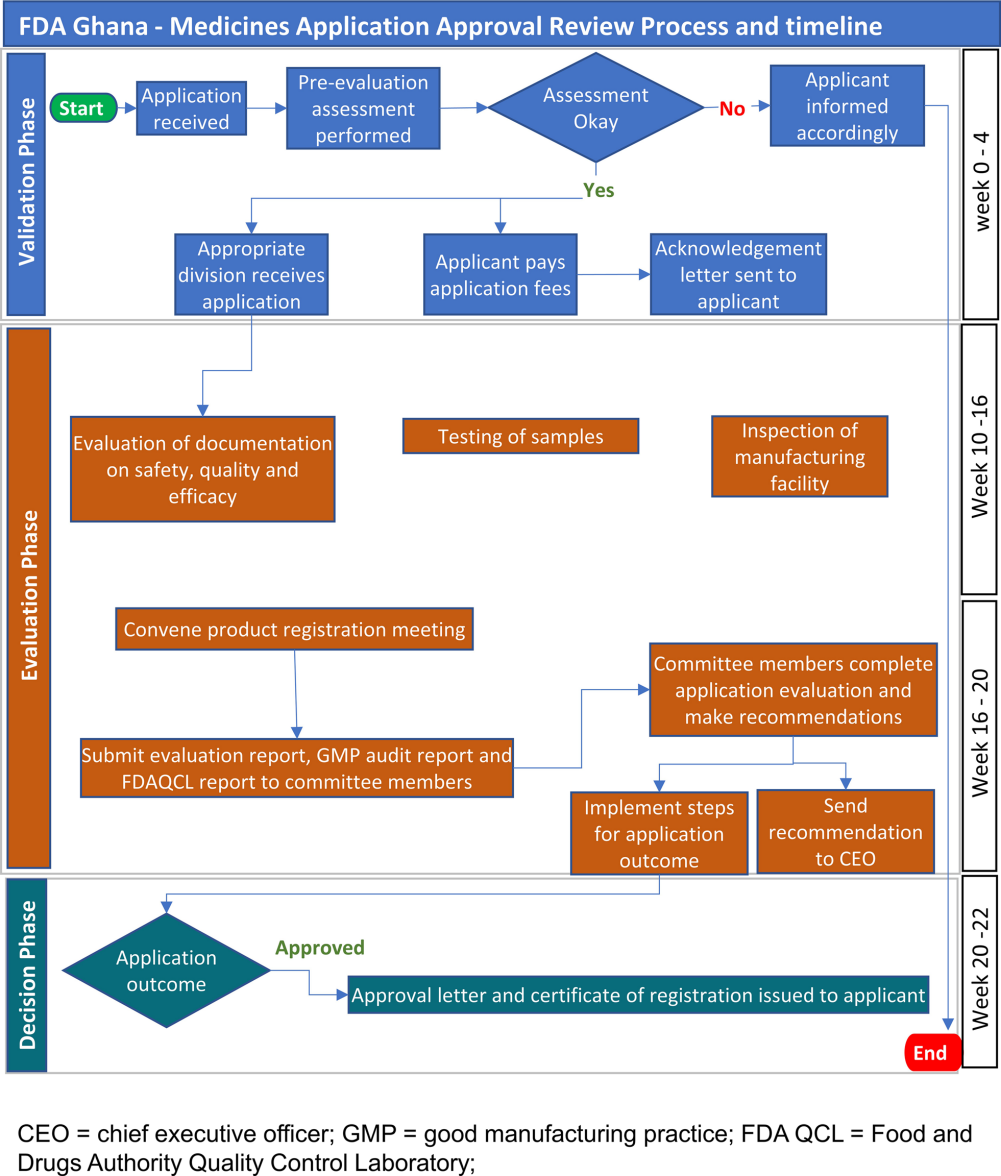
Regulatory review process map for Ghana showing target times in calendar days; representing the review and authorisation of a product that goes to approval after one review cycle.

A map of the review process and timelines for applications by the FDA Ghana is provided (Fig. 1) showing the three phases in the review process, namely validation, evaluation and decision. The review process is presented in a format that correlates with the key milestones in the review procedure. It should be noted that the process map is a simplified representation of the main steps in the full review of an application and represents the review and authorisation of a product that is approved on the first cycle. The map does not include a second cycle for products approved subject to the submission of additional data nor does it include the steps that follow the refusal of an application, such as hearings or appeals.

#### Validation Phase

Within a month of receipt of the submission in the common technical document (CTD) format for marketing authorisation, the application is validated for completeness and acceptance is formally recorded. A new application is held in the queue before the start of scientific assessment. Priority products are, however, taken out of the queuing system. Applications are assessed on a first-in first-out (FIFO) basis unless the product meets the classification criteria for expedited review process as set out in the FDAs guidelines for registration of pharmaceutical products.

An application is classified as priority and may be expedited if the product is for any of the following; public health programmes (including HIV/AIDS, malaria, tuberculosis, reproductive health, neglected tropical diseases or an expanded programme of immunisation), paediatrics, Ministry of Health tender purposes, WHO prequalification-collaborative registration process and any other disease or conditions as may be determined by the FDA from time to time). The timeline for processing priority applications is three months. The FDA Ghana does not regard the backlog of applications as a problem, as the technical capacity of assessors enables them to process applications efficiently.

#### Evaluation Phase

The scientific assessment is carried out by technical staff of the authority who are assigned to review the quality, safety, and clinical documentation. Questions are collected into a single batch and sent to the sponsor. An applicant can hold meetings with the authority staff following the receipt of questions from the authority that arise during the assessment. There is no “clock stop”, therefore the overall review and decision time includes the time taken by the applicant to respond. The evaluation of the dossier (documentation on safety, quality and efficacy), laboratory analysis of samples and inspection of the manufacturing facility are conducted, and the respective reports are presented to a high-level committee, referred to as the Drug Registration Committee, for review. The Drug Registration Committee meets each month and makes final decisions to grant or refuse marketing authorisations.

#### Decision Phase

This authorisation procedure is dependent on sample analysis and inspection of the manufacturing facility, but these are conducted in parallel with the scientific review and therefore do not delay the decision of marketing authorisation. The procedure is duly completed following the issuance of a certificate of registration for the product.

### Summary of Applications Registered from 2019–2021

There was a successive annual increase in the number of products approved over the three-year period from 177 in 2019 to 236 in 2020 and to 362 in 2021. The observed trend is mainly attributed to an 80% reduction of marketing authorisation application fees in January 2020 to $240, $360 and $300 per annum for generic medicinal products, new chemical entities and biological products, respectively. Along with the 80% decrease in application fee, a verification fee of 0.80% of the CIF (Cost, Insurance and Freight) value was introduced for imported regulated products. Applicants preferred this option since they only had to pay a comparatively small application fee at the time of applying for marketing authorisation and then pay the verification fee at the time of importing each consignment of the product into the country. More importantly, the Authority is able to increase its revenue with this approach. This is therefore a win–win strategy for the Authority and industry. This has enabled sponsors to submit more applications at a lower cost and consequently resulted in an increase in the number of marketing authorisations granted.

### Characteristics of New Active Substances Registered Between 2019 and 2021

During the period 2019–2021, 67 NASs (comprising 61 (91%) biologicals and six (9%) pharmaceuticals) were registered by the FDA Ghana (Table 1). Whilst 22 NASs (comprising 20 biologicals and 2 pharmaceuticals) were registered in 2019 and 26 (comprising 22 biologicals and 4 pharmaceuticals) in 2021, there was a reduction to 19 (all biologicals) in 2020 (Table1), this was apparently due to the pandemic. Furthermore, in 2020, no Covid-19 pandemic-related products were authorised; however, of the 19 NASs authorised in 2021, 11 (10 biological and one pharmaceutical) were Covid-19 pandemic-related.

**Table 1:** Characteristics of new active substances approved between 2019 and 2021

Characteristic	Approval year			
	2019	2020	2021	2019–2021
Number of new active substances				
Overall	22	19	26	67
Review type				
Verification	3	0	1	4
Abridged	3	7	14	24
Full	16	12	11	39
Priority review				
Yes	3	7	13	23
No	19	12	13	44
Not specified	0	0	0	0

The majority (39) were reviewed using the full review model, 24 by the abridged and only 4 by the verification review pathway. The number of abridged reviews increased from 3 in 2019 to 14 in 2021, whilst full reviews were reduced from 16 in 2019 to 11 in 2021. During the same period, the NASs that were subject to priority review increased from 3 in 2019 to 13 in 2021. Of the NASs, 91% were biologicals and were processed by the full review and abridged reviews pathways with relatively longer review times when compared with generics, although the review times for processing these NASs were within the target decision timeline of 38 weeks (266 calendar days).

### Characteristics of Generics Registered Between 2019 and 2021

During the period 2019–2021, a total of 708 generic products (comprising 18 (3%) biologicals products and 690 (97%) pharmaceuticals) were registered by the FDA Ghana (Table 2). There was an increase from 155 in 2019 to 217 in 2020 and 336 in 2021 (Table 2). The majority of these generics (149 in 2019 to 322 in 2021) were the subject of full review, with only three generics approved by the abridged pathway, all in 2021. During this period, 25 generics were approved by the verification pathway, six in 2019, eight in 2020 and 11 in 2021. It was reported that very few generic products (92) were given a priority review, although this increased from 15 in 2019 to 55 in 2021, suggesting a pandemic impact. In general, the generics reviewed by the verification pathway were for the treatment of HIV/AIDS, malaria, tuberculosis, diarrhoea, COVID-19-related therapies and reproductive therapeutics. Of the generics, 97% were pharmaceuticals and were processed by the full review pathway with relatively shorter review times when compared to NASs, and the approval times for processing these generics were within the target timelines. The review types and numbers reflect the large volumes of generic applications compared with NASs originating from low- and middle-income countries (LMICs) [14].

**Table 2:** Characteristics of generics approved between 2019 and 2021.

Characteristic	Approval year			
	2019	2020	2021	2019–2021
Number of generics				
Overall	155	217	336	708
Review type				
Verification	6	8	11	25
Abridged	0	0	3	3
Full	149	209	322	680
Priority review				
Yes	15	22	55	92
No	140	195	280	615
Not specified	0	0	1	1

### Overall Decision Timelines for Registered Products

**Figure 2:**
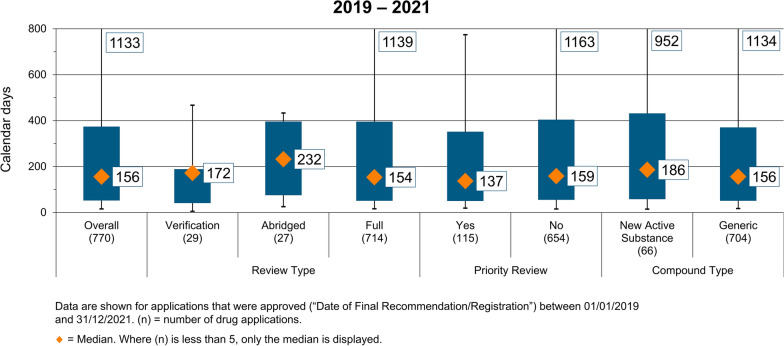
Overall decision times for all products 2019–2021

The overall timelines for all products (combined NASs and generics) over the period 2019–2021 are shown in Fig. 2. During this period, the median approval time ranged from 137 to 232 calendar days. This demonstrates the range in approval times, with the diamond representing the median value, the box the range between the 25th and the 75th percentile, whilst the whiskers represent the outliers, which are the 5th and 95th percentiles. This visual representation fully describes the regulatory burden for the FDA Ghana. During this period, 770 products were approved, with a median time of 156 calendar days. Not surprisingly, the median value for the 704 generic products was also 156 calendar days, whilst the median value for the 66 NASs was 186 calendar days. These review times were within the target approval timeline of 266 calendar days. (This includes both the agency and the industry response time, as per the FDA Ghana website.)

### Overall Decision Timelines for New Active Substances

**Figure 3:**
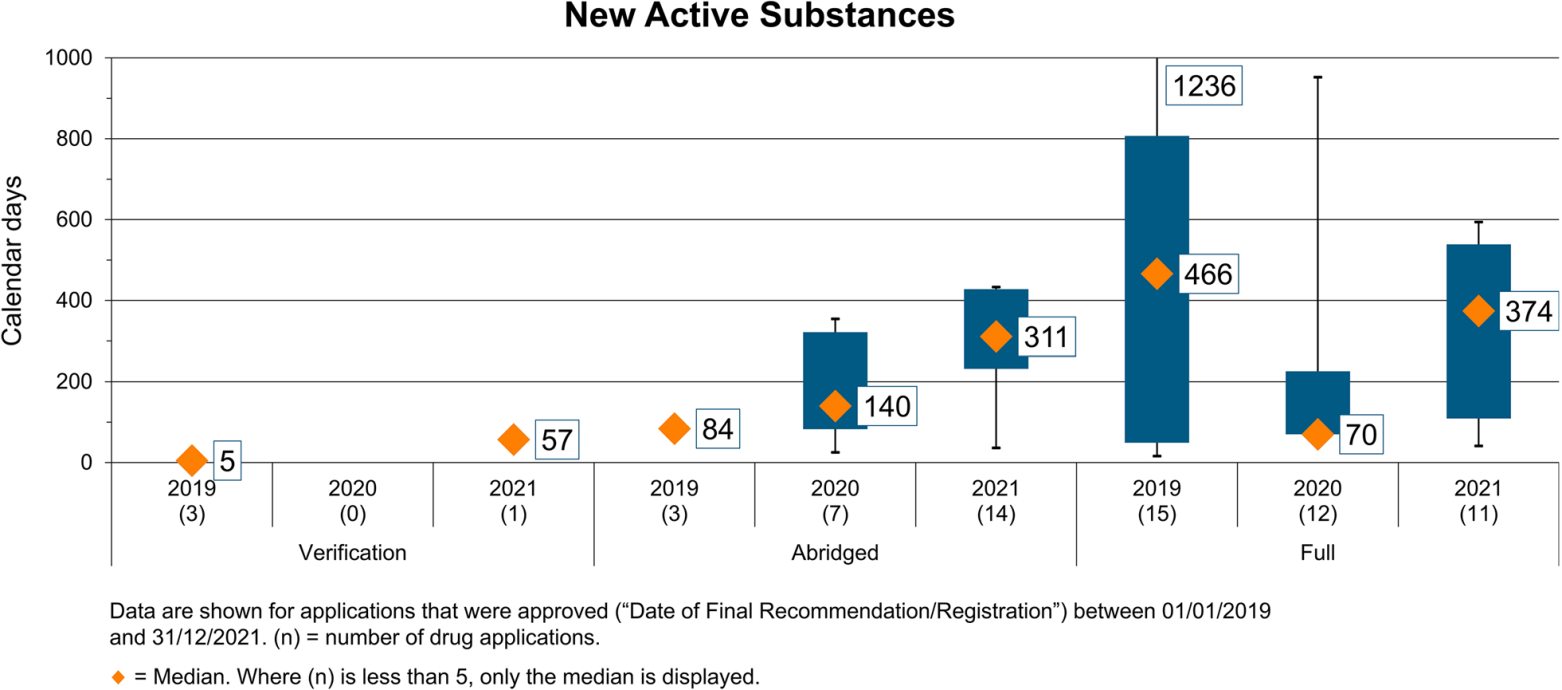
Overall decision times for new active substances 2019–2021.

The overall decision time for NASs registered between 2019 and 2021 (comprising 61 (91%) biologicals and six (9%) pharmaceuticals) is displayed in Fig. 3. During this period, the median decision times for full review ranged from 466 calendar days (15 NASs) in 2019 to 70 calendar days (12 NASs) in 2020, with 374 calendar days (11 NASs) in 2021. It is of interest to note that during 2019, the range was extensive (i.e. 50 to 800 calendar days), which is substantially different from the range in 2020 and 2021. Since the time used for scientific assessment/review contributes to the overall decision time, analysis of the overall decision time indicated that all the 15 NASs that were registered in 2019 were biologicals; the median for scientific assessment with regard to full review of these NASs conducted in 2019 was 138 days, with a range of 40 to 170 days. It can therefore be seen that the long decision time was due to the time taken by applicants/ sponsors to respond to the request for additional data.

The increase in the number of the NASs approved may have contributed to the increase in decision times. In general, the decision time by the abridged route was quicker than the full review pathway, with the exception of the year 2020, when the median for full review was 70 calendar days, whereas the median for the abridged review of NASs was 140 calendar days. This difference is apparently due to applicant response time. Therefore, it is recommended that the agency should implement a robust IT system to help it separate agency time and applicant time appropriately.

**Figure 4:**
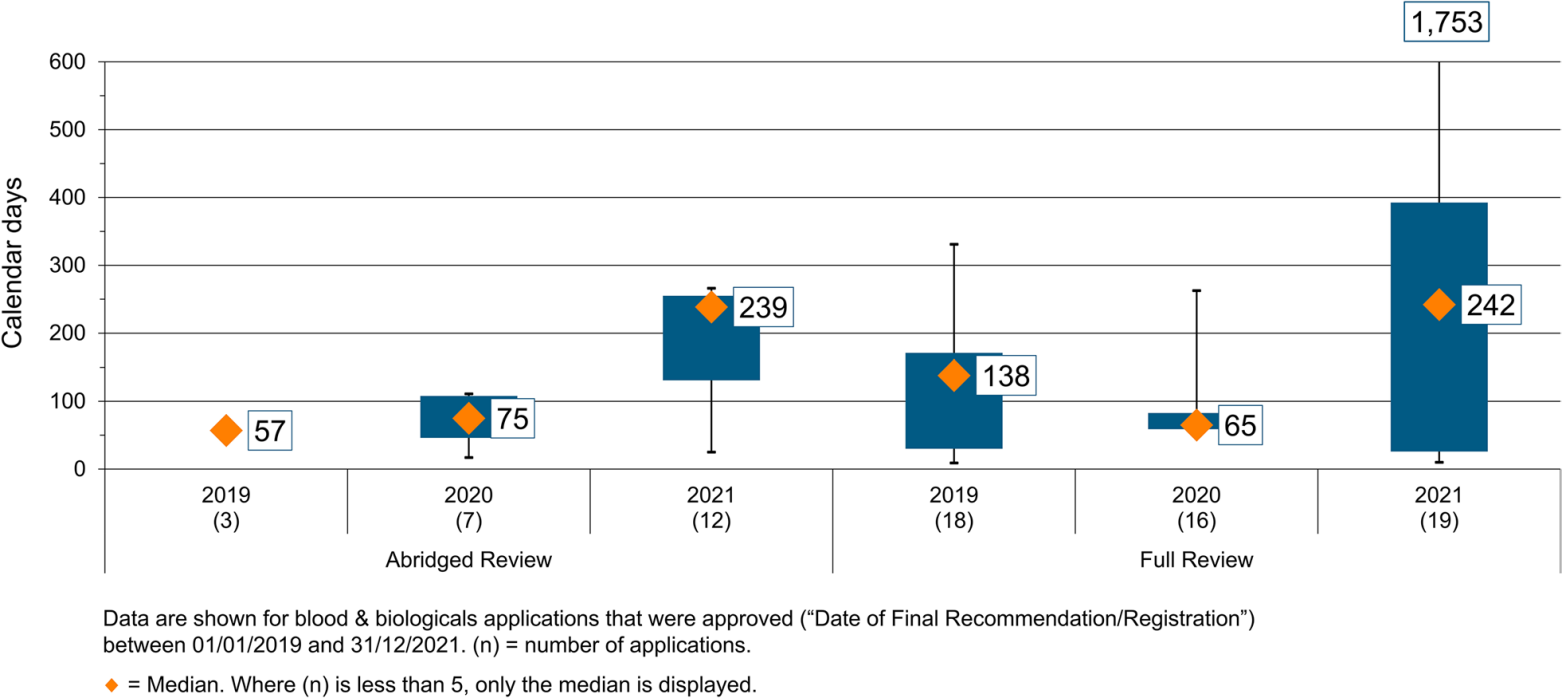
Scientific assessment time for full review of biologicals 2019–2021.

The scientific assessment time taken by the FDA Ghana to conduct product registration meetings as part of the approval process and the time taken to complete administrative processes is shown in Fig. 4. The median for scientific assessment with regard to full review of NASs, the majority of which were biological products, was 65 days (range, 60 to 80 days) in 2020 (Fig. 4). However, the median for scientific assessment with regard to the abridged review was 75 days (range, 50 to 110 days) in 2020 (Fig. 4).

It can be noted that although the time taken for scientific assessment was similar for both full and abridged reviews, approval time for abridged reviews, which were reported as double that for full reviews in 2020 were due to the relatively longer time taken by applicants/sponsors to respond to request for additional data. This was the result of the pandemic, when unusually long review times and delays were considered to be as expected. Moreover, very few NASs were approved using the verification pathway during that time (Fig. 3).

During this period, the median approval times for abridged review ranged from 84 calendar days in 2019 to 311 calendar days in 2021. Furthermore, in 2019 only 3 NASs (all biologicals) were approved using the abridged facilitated regulatory pathway, and 7 (all biologicals) in 2020, which increased to 14 (12 biologicals and 2 pharmaceuticals) in 2021. The median for scientific assessment in the abridged review of these NASs was 57 days in 2019, 75 days (range 50 to 110) in 2020, and 239 days (range 130 to 250) in 2021.

Similarly, with full review, the time taken by applicants/sponsors to respond to request for additional data, the time taken by the FDA Ghana to conduct product registration meetings as part of the approval process and also the time taken to complete administrative processes at the FDA Ghana had an effect on the approval times (Fig. 4).

### Overall Decision Timelines for Generics

**Figure 5:**
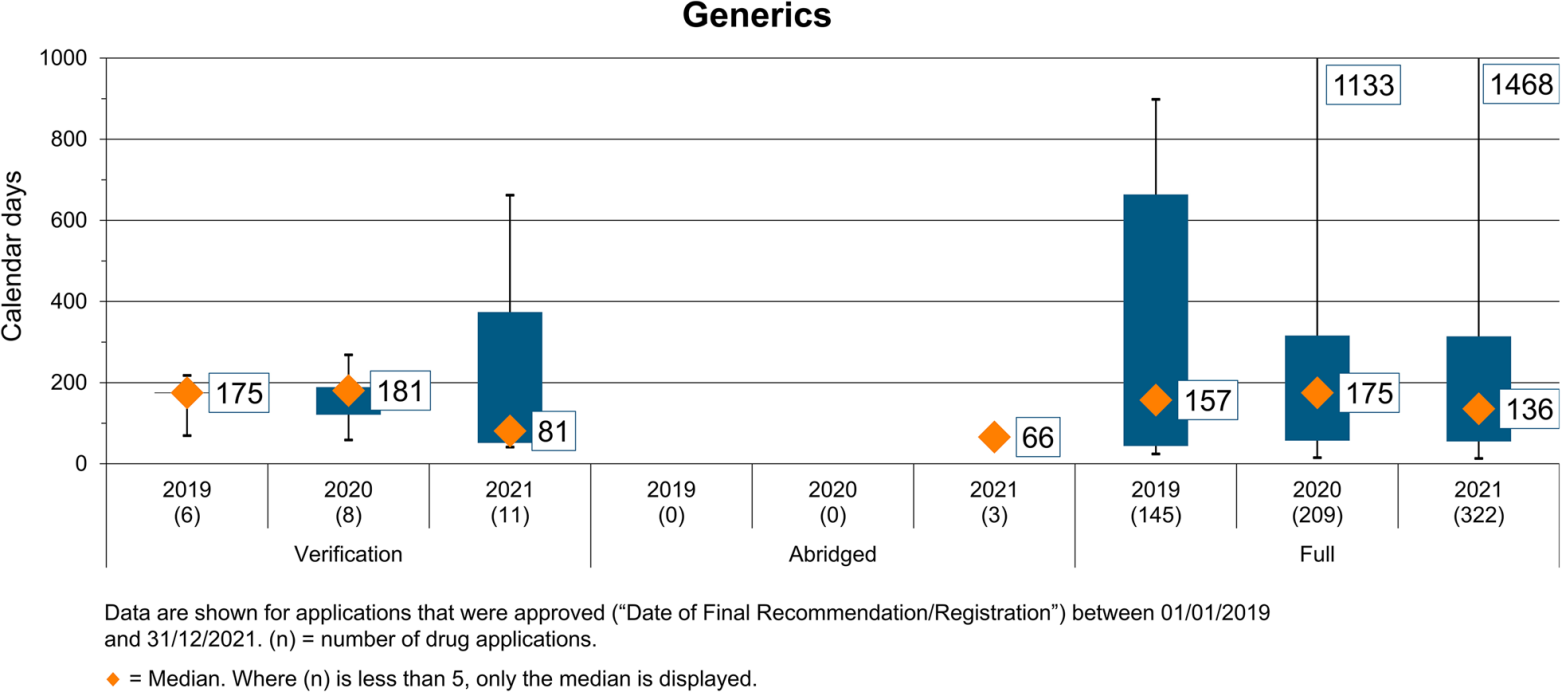
Overall decision times for generics 2019–2021.

The overall decision time for generic products during the period 2019–2021 encompassing the three different regulatory pathways (verification, abridged and full) is shown in Fig. 5. The majority of products were subject to full review within consistent decision times of 157 calendar days in 2019 (145 generics) and 175 calendar days in 2020 (209 generics) and reduced to 136 calendar days (322 generics) in 2021. The range in decision times, which was substantial in 2019, was reduced considerably in 2020 and 2021. It is of interest to note that coincidentally with reduction in decision times between 2019 and 2021 there was more than doubling in the number of products registered (145–322, respectively).

During this period, very few generics were registered using verification pathway; that is, 6 in 2019, 8 in 2020 and 11 in 2021. The median decision time for these products ranged from 175 calendar days (2019) to 181 calendar days (2020) and was reduced to 81 calendar days in 2021. Over this period, only three generics were reviewed using the abridged pathway, with a median approval time of 66 calendar days. It is worth noting that whilst the median decision time for full review was consistent during this period, there were significant outliers of 1133 and 1468 calendar days due to applicant response time to questions, which indicates an opportunity for improvement. It is of interest to note that those generics undergoing full assessment were doing so as a result of choosing not to go through the PQ process and included both locally produced and imported products.

### Part 4—Good Review Practices (GrevP)—Building Quality into the Regulatory Process

The authority has implemented some quality measures in the review and authorisation of medicinal products as summarised in Table 3.

**Table 3:**
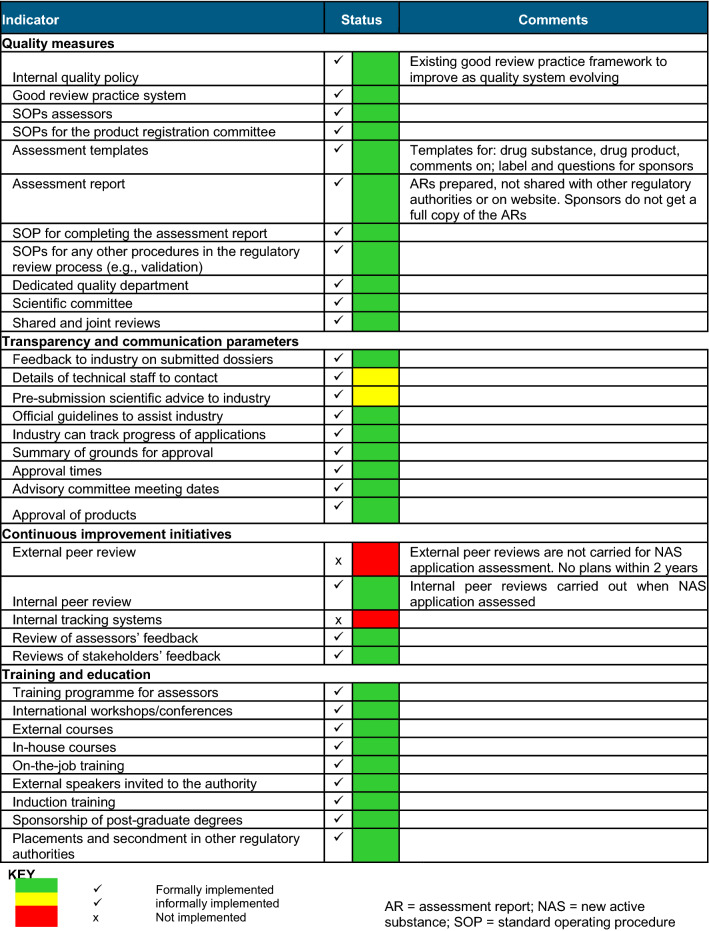
Status of implementation of good review practices by the FDA Ghana

### Quality and Transparency Measures

Ensuring quality and transparency in a pharmaceutical regulatory system improves patients’ access to quality, safe and effective medicines [15]. FDA Ghana identified three important measures as necessary for the management of quality, and these include measures for ensuring consistency and increasing transparency, and achieving stakeholder satisfaction. The FDA Ghana achieved International Organization for Standardization (ISO) 9001:2015 certification in June 2017, affirming the FDA Ghana commitment to meeting international process standards in order to help provide quality products and services. This certification is assiduously maintained by the authority and there is a dedicated department with staff involved in assessing and/or ensuring quality in the registration process, which is carried out annually under the supervision of the Deputy Chief Executive Officer. This implies achieving system-wide effectiveness and efficiency using cutting-edge technology as an enabler***.***

In order to improve the quality of applications and the scientific review, the following measures have also been implemented:

- Official guidelines to assist the industry are available, in English, through the authority website and on request by stakeholders.
- Pre-application scientific advice is given to applicants and discussions are held with reference to the applicable guidelines, which ensures consistency in the information shared with applicants. Applicants are encouraged to engage with the authority early in the product development process to ensure that there is clarity on needed data points and components in the dossier.
- A pool of internal assessors is available to review dossiers and to provide detailed assessment reports, clinical opinions on the product and technical advice to the authority.
- The Drug Registration Committee, which is an internal committee, in turn reviews all applications by reviewing the assessment reports, GMP audit reports and sample testing and makes decisions on the granting of marketing authority to the authority.

The authority participates in the West African Health Organization (WAHO) regional harmonisation initiative and has conducted shared or joint reviews with other regulatory authorities. There are formal measures in place to ensure consistent quality during the review through the WAHO Joint Assessment and this work-sharing process has had a positive impact on the work of the authority in general. In addition, bilateral and multilateral information-sharing agreements are in place with other jurisdictions with a collaborative procedure and are part of participation in the WHO Pre-Qualification procedure and the WHO PQ-NMRA Collaborative Review Process.

The authority assigns high priority to being open and transparent in its relationships with the public, health professionals and the pharmaceutical industry. The authority is driven by three incentives for assigning resources to activities that enhance the openness of the regulatory system. This includes the need to provide assurances on safety safeguards, to increase confidence in the system and to efficiently meet and address the healthcare of the population. The FDA Ghana informs the general public about authority regulatory activities by providing information on approved products on their website. Companies can follow the progress of their applications by telephone and e-mail contact, and they are also given detailed reasons for rejection of their applications. There is no electronic system for registering and tracking sponsor applications; however, there are plans to introduce such a system by the end of 2022.

#### Continuous Improvement Measures

The FDA Ghana has addressed the training and continuing education needs of assessors by modelling WHO recommendations that have been adopted by the EMA and the Federal institute for drugs and medical devices (Bfarm), often providing training in collaboration with other mature agencies. The authority acknowledges the importance of having measures in place to continually improve the review process [16] and one important strategic measure is to ensure that assessors acquire international technical expertise to order to process applications in an efficient manner. The authority also participates in international workshops and training programmes [17].

### Part 5—Quality Decision-Making Practices

The FDA Ghana has implemented guidelines and tools that enables it to have structured approach to quality decision-making practices. The roles and responsibilities of the regulator, manufacturers and national and international stakeholders have been defined and communicated on the authority website. The authority implements certain aspects of the quality decision-making practice framework as the basis to approve or reject a marketing authorisation application, as summarised in Table 4.

**Table 4:**
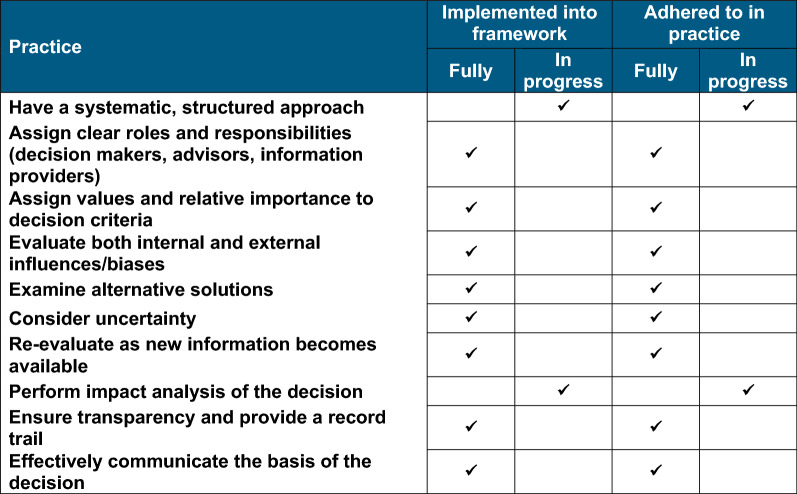
FDA Ghana quality decision-making practices

### Part 6—Concluding Observations

The effectiveness and efficiency of the FDA Ghana review procedures and decision-making practices for medicinal product applications are enhanced by the continuous professional training of staff and the continuous internal audit of review processes as well as the development of published timelines for all the critical stages of the review. However, insufficient data for a product, unsatisfactory GMP compliance or substandard dossier submission can inhibit the timely approval of medicinal products by the authority.

## Discussion

The WHO has recently reported that globally only about 30% of medicine regulatory authorities are performing to the basic, minimal standard expected of a regulatory authority. In view of this, the WHO is exploring various solutions to address this problem. One of these is the introduction of a WHO-listed Authorities (WLA) programme for regulatory authorities. When fully instituted—after an interim transitional period of 5 years—it will apply to NRAs who have achieved an overall ML3 accreditation by WHO (this is required to be eligible for WLA consideration) and who have, in addition, achieved ML4 either overall or in specific Global Benchmarking Tool modules for which the NRA wishes to be recognised as an WLA. Finally, the NRA will need to have demonstrated its ability to maintain this level of performance to WHO satisfaction for a stated period of time. When fully implemented, this will signal to the global community that such WLA agencies are those on whom agencies can rely as reference agencies with confidence, if they choose. Currently five countries in Africa (Egypt, Nigeria, Ghana, South Africa, and Tanzania) have medicine regulatory agencies that have reached ML3 status (i.e. eligible for WLA, when the programme is fully implemented) [18].

The authority employs the three established regulatory review models for assessing marketing authorisation applications. The extent to which quality, safety and efficacy data are assessed depends on the review model. The first and final milestones dates in the review process are the receipt of the application and the registration approval date. Currently, there is not as yet an electronic tracking system in place and therefore the obvious challenges associated with a manual system are evident in the data collection processes. The FDA Ghana is taking steps to build quality into the regulatory process but has not yet started publishing Public Assessment Reports on its website. It is hoped that publishing these assessment reports, including steps taken in the assessment process, will provide details on the time spent at each milestone of the process. After this, recommendations for ways to address possible delays in the review process can be implemented to achieve the overall regulatory goal of enhancing patients’ access to quality, safe and efficacious medicines.

If manufactured and used appropriately, generic medicines can have major medical and economic benefits for the healthcare of a nation. It has been reported that generic medicines constitute about 90% of prescriptions in the USA and this has reduced healthcare cost by 2.2 trillion dollars as a result of using generics instead of new chemical entities [20]. This study has demonstrated that generic medicines (including biosimilars) constituted 91% of medicines approved by the FDA Ghana from 2019 to 2021. These medicines are processed faster than NASs, mainly because of their relatively simpler clinical requirements. FDA Ghana has also developed adequate technical capacity to assess these generic applications. Due to the demand for generic medicines in LMICs, most NMRAs dedicate significant resources to evaluate applications for marketing authorisations quickly so that the healthcare system can enjoy these cost-saving benefits [14]. Additionally, these generics products can often be assessed by pharmacists rather than physicians (bioequivalence and manufacturing quality) as is reflected in the FDA Ghana, where 25 of the 26 internal reviewers are pharmacists.

It was also reported that the average time between generic drug application submission and approval in the USA was about 6 months and 10 months for priority review and standard review, respectively [14, 20]. The approval timeline for generics was 175 working days and 180 calendar days for Australia and Canada, respectively [14, 20]. Therefore, the median approval times for generics approved in Ghana, which was in the range of 81 to 181 calendar days, were comparable to the approval timelines in the USA, Australia and Canada.

## Recommendations

The following recommendations for FDA Ghana were identified from the study:

- Product-specific guidelines should be provided to help applicants comply with the registration requirements and obtain approval after one review cycle.
- An electronic tracking system should be implemented to enable the authority and applicants to track applications for marketing authorisations.
- Annual training workshops should be arranged for manufacturers to help them with submission of fully completed dossiers to facilitate the review process and decrease approval timelines.
- Efficient ways should be explored to review marketing authorisation applications for NASs that are assessed via the full review pathway.
- A comparison with other stringent regulatory authorities should be carried out to identify best practices.
- Public assessment reports for all marketing authorisation applications should be made available.
- A systematic and well-structured quality decision-making practice framework should be implemented.
- The timelines for review and decision on a product should be established in terms of both the agency and the industry time.

## Abbreviations

BfArM: Federal institute for drugs and medical devices
CIRS: Centre for innovation in regulatory science
CPP: Certificate of pharmaceutical product
CTD: Common technical document
EDQM: European directorate for the quality of medicines & healthcare
EMA: European medicines agency
GBT: Global benchmarking tool
GMP: Good manufacturing practice
GRevP: Good review practices
ICH: International council for harmonisation of technical requirements for pharmaceuticals for human use
LMICs: Low- and middle-income countries
MHRA: Medicines and healthcare products regulatory agency
NASs: New active substances
NMRA: National medicines regulatory authority
OpERA: Optimizing efficiencies in regulatory agencies
QMS: Quality management systems
SRAs: Stringent regulatory authorities
US FDA: United States food and drug administration
WHO: World Health Organization
WLA: WHO-listed authorities
